# Glycosylation of *Staphylococcus aureus* cell wall teichoic acid is influenced by environmental conditions

**DOI:** 10.1038/s41598-019-39929-1

**Published:** 2019-03-01

**Authors:** Noëlle Mistretta, Marina Brossaud, Fabienne Telles, Violette Sanchez, Philippe Talaga, Bachra Rokbi

**Affiliations:** Research and Development, Sanofi Pasteur, Marcy l’Etoile, France

## Abstract

Wall teichoic acid (WTA) are major constituents of *Staphylococcus aureus* (*S*. *aureus*) cell envelopes with important roles in the bacteria’s physiology, resistance to antimicrobial molecules, host interaction, virulence and biofilm formation. They consist of ribitol phosphate repeat units in which the ribitol residue is substituted with D-alanine (D-Ala) and N-acetyl-D-glucosamine (GlcNAc). The complete *S*. *aureus* WTA biosynthesis pathways was recently revealed with the identification of the two glycosyltransferases, TarM and TarS, respectively responsible for the α- and β-GlcNAc anomeric substitutions. We performed structural analyses to characterize WTAs from a panel of 24 *S*. *aureus* strains responsible for invasive infections. A majority of the *S*. *aureus* strains produced the β-GlcNAc WTA form in accordance with the presence of the *tarS* gene in all strains assessed. The β-GlcNAc anomer was preferentially expressed at the expense of the α-GlcNAc anomer when grown on stress-inducing culture medium containing high NaCl concentration. Furthermore, WTA glycosylation of the prototype *S*. *aureus* Newman strain was characterized *in vivo* in two different animal models, namely peritonitis and deep wound infection. While the inoculum used to infect animals produced almost exclusively α-GlcNAc WTA, a complete switch to β-glycosylation was observed in infected kidneys, livers and muscles. Overall, our data demonstrate that *S*. *aureus* WTA glycosylation is strongly influenced by environmental conditions and suggest that β-GlcNAc WTA may bring competitive advantage *in vivo*.

## Introduction

*Staphylococcus aureus (S*. *aureus)* is frequently a commensal symbiont in the human host, and one of the most successful opportunistic pathogens causing severe infections worldwide. The bacteria has acquired the ability to manipulate and evade host immune surveillance and responses^1,2^. Infection with *S*. *aureus* can cause a range of symptoms, from relatively minor skin abscesses/boils to fully disseminated disease including, endocarditis, sepsis and toxic shock syndrome. Invasive disease is associated withc ≥20% mortality rate^3^.

*S*. *aureus* is surrounded by cell surface polysaccharides, including capsular polysaccharides (CPs) and teichoic acids (TAs)^4^. There are two types of TAs: lipo-TAs (LTA), which are anchored in the cytoplasmic membrane, and cell wall TAs (WTAs), which are covalently linked to peptidoglycan in the bacterial cell wall.

WTAs contribute to staphylococcal adhesion and colonization^5,6^, and play a role in cell division and biofilm formation^7^, and their overexpression increases *S*. *aureus* virulence^8^. In addition, D-alanine (D-Ala) residues on TAs contribute to resistance to cationic antimicrobial peptides such as defensins or cathelicidins, and to glycopeptide antibiotics such as vancomycin or teicoplanin^9–11^.

There is substantial variation in the chemical structure of WTAs among Gram-positive bacteria^12^. *S*. *aureus* WTAs consist of poly(ribitol-phosphate) substituted at the O-2 and O-4 positions of the ribitol residue with D-Ala and α- or β-N-acetylglucosamine (GlcNAc), respectively^13–15^. *S*. *aureus* WTAs have been considered as putative vaccine candidate^4,16^. The pioneer work in that field was conducted by Nabi Biopharmaceuticals with Antigen 336 (Ag336), also named Polysaccharide 336 (PS336). Ag336 was purified from a strain deposited at ATCC under number ATCC 55804 and used to serotype *S*. *aureus* isolates that do not express capsule^17^. Ag336 was reported as a cell surface polysaccharide consisting of ribitol, GlcNAc and phosphate, and hence reveals to be WTA. However, in contrast to conventional WTA the ribitol residue of Ag336 is substituted at the O-3 and not O-4 position with β-GlcNAc^18^.

Although *S*. *aureus* WTA glycosylation with α- and β-GlcNAc was reported in the 1960s, the glycosyltransferases responsible, TarM and TarS, were only identified recently^19,20^, While TarM is α-glycosyltransferase, TarS is a β-glycosyltransferase that glycosylates the O-4 of ribitol respectively. The *tarS* gene is present in near all sequenced *S*. *aureus* genomes, with very few exceptions, whereas *tarM* is absent in a number of strains such as strains of CC5 and the emerging clonal complex CC398^21^. In general, the presence or absence of the *tarS*, and *tarM* genes in *S*. *aureus* strains has been used to infer their WTA glycosylation pattern^19,21–24^. While the presence of the *tarM or tarS* genes can be easily detected by polymerase chain reaction (PCR), high-throughput structural analyses of WTA, and more specifically WTA glycosylation, remain challenging. Purified WTA samples and nuclear magnetic resonance (NMR) spectroscopy are required, rendering the identification of glycosylation pattern both laborious and time-consuming.

In addition, the potential of individual *S*. *aureus* strains to regulate the expression of both α- and β-GlcNAc anomers under different environmental conditions has not been assessed.

Herein, we report structural analyses by a High-Performance Anion-Exchange Chromatography (HPAEC)-based method that has allowed us to characterize WTAs from a panel of 24 *S*. *aureus* strains responsible for invasive infections, grown *in vitro* under normal and stress-inducing culture conditions. We show that a majority of the *S*. *aureus* strains produced the β-GlcNAc WTA form, which is consistent with the presence of *tarS* gene in most of the strains, and we present evidence that *S*. *aureus* is able to modify the phenotype of its WTA glycosylation pattern by exposure to environmental stress-inducing culture conditions. Furthermore, we report for the first time the characterization of WTA directly on infected mouse tissues without any purification or bacteria culture step, and we show a preferential switch to β-glycosylation of WTAs i*n vivo* using two mouse infection models, with the Newman strain as prototype strain.

## Results

### The panel of strains selected is representative for *Staphyloccus aureus* clinical diversity

In order to study the extent of variation of WTA composition among *S*.*aureus* strains, a panel of 24 strains isolated from 1905 to 2005 was selected (Table 1). The genetic diversity and representativeness of the panel was firstly illustrated by the presence of the four accessory gene regulator *agr* groups. The *agr* locus controls the expression of genes for virulence factors that play a major role in the virulence of *S*. *aureus*. Expression of these genes is controlled by the accessory gene regulator (*agr)* locus^25,26^. This locus is recognized as a quorum-sensing system in *S*. *aureus*^27^. Based on polymorphisms in *agrB*, *agrC* and *agrD* genes, four major allelic *agr* groups (I, II, III and IV)^27,28^ have been characterized, each associated with specific staphylococcal disease^25^. Secondly, as the adaptation of *S*. *aureus* to the environment has been marked by the acquisition of methicillin-resistance, the collection included both MRSA and MSSA strains as predicted by the detection of the *mecA* gene^29^. Thirdly, the chosen panel included the two major capsular genotypes (5 and 8) among *S*. *aureus* isolates causing human infections^30,31^. Fourthly, the strains are representative of the various types of infection as they were isolated from patients with eight different diseases. Finally, we characterized the strains for the presence of WTA glycosyltransferase *tarS* and *tarM* genes. All the strains displayed the *tarS* gene. The *tarM* gene was present in 11 out of 24 strains representing 45.8% of the panel. Moreover the sequence of *tarS* and *tarM* were determined for strains HH0528 1156, HT2005 0756, HT2005 0843 and was found to be identical to the reference sequences.

**Table 1:** *Staphylococcus aureus* strains used in the study.

Strain name	geographic	isolation	type	genetic characterization*					Source
	origin	date	of infection	*cap*	agr type	*mecA*	*tarS*	*tarM*	
HT2005 0667	France	2005	septic shock	5	2	S	+	−	HEH, Lyon
HT2005 0769	France	2005	pneumonia	5	2	S	+	−	HEH, Lyon
HT2005 0699	France	2005	osteomyelitis	5	2	R	+	−	HEH, Lyon
HT2005 0749	France	2005	toxic shock	5	2	R	+	−	HEH, Lyon
HT2005 0847	France	2005	skin	5	2	S	+	−	HEH, Lyon
HT2005 0659	France	2005	diarrhea	5	2	S	+	−	HEH, Lyon
HT2005 0742	France	2005	septic shock	5	1	S	+	−	HEH, Lyon
HT2005 0499	France	2005	respiratory	5	1	S	+	−	HEH, Lyon
HT2005 0662	France	2005	foodborne	5	nd	S	+	−	HEH, Lyon
HT2005 0704	France	2005	skin	8	4	R	+	+	HEH, Lyon
HT2005 0828	France	2005	skin	nd	3	S	+	+	HEH, Lyon
HT2005 0702	France	2005	skin	5	4	S	+	+	HEH, Lyon
HT2005 0689	France	2005	osteomyelitis	5	1	R	+	+	HEH, Lyon
HT2005 0756	France	2005	skin	5	1	R	+	+	HEH, Lyon
HT2005 0837	France	2005	septic shock	5	1	S	+	+	HEH, Lyon
HT2005 0726	France	2005	osteomyelitis	8	2	S	+	+	HEH, Lyon
HT2005 0843	France	2005	skin	5	1	R	+	+	HEH, Lyon
HH0528 1156	France	2005	septic shock	5	nd	R	+	+	HEH, Lyon
ATCC 55804	Unknown	Unknown	urinary	5	nd	S	+	−	ATCC
Newman	UK	1952	osteomyelitis	5	nd	S	+	+	ATCC
Reynolds	USA	1982	septic shock	5	nd	S	+	−	CIP
SA113	UK	1943	unknown	5	nd	S	+	+	ATCC
Wood46	Australia	1905	skin	5	nd	S	+	−	ATCC
Wright	USA	1979	bacteremia	8	nd	S	+	−	CIP

### A chromatographic method was developed to rapidly determine WTA structures

**Figure 1:**
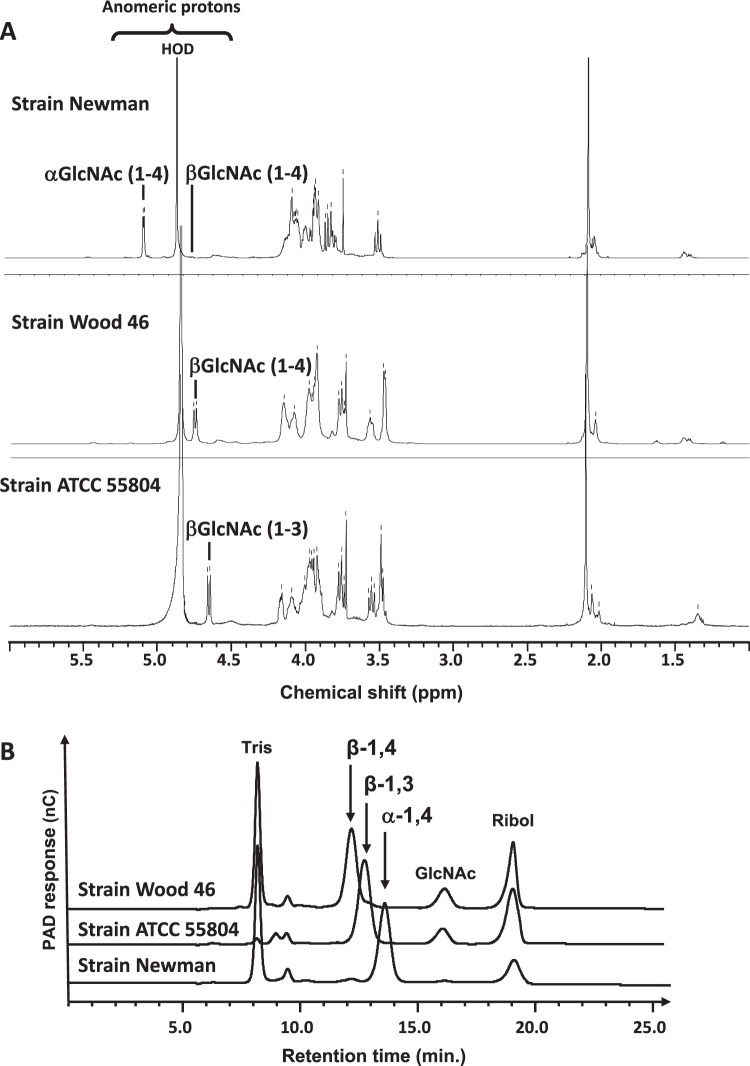
Glycosylation pattern of the Newman, Wood 46 and ATCC55804 WTAs. (**A**) 500 MHz ^1^H NMR spectra of purified WTAs in D_2_O at 293 K; (**B**) HPAEC-PAD chromatograms of GlcNAc-ribitol disaccharides obtained from *S*. *aureus* WTA HF-hydrolysis of the Wood 46 strain (β-D-GlcNAc-(1→4)-ribitol), ATCC55804 strain (β-D-GlcNAc-(1→3)-ribitol) and Newman (α-D-GlcNAc-(1→4)-ribitol) strains. GlcNAc: N-acetyl-glucosamine, Ribol: Ribitol, Tris: Tris buffer in samples.

WTAs were extracted and purified from the Newman, Wood 46 and ATCC55804 strains grown in a complex commercially available medium (tryptic soy broth, TSB). Analysis of the monosaccharide composition of the WTAs by High-Performance Anion-Exchange Chromatography with Pulsed Amperometry Detection (HPAEC-PAD) showed the presence of ribitol and glucosamine. It should be noted that GlcNAc is transformed to Glucosamine (GlcN) under the conditions used for WTA hydrolysis. The GlcN/Ribitol molar ratio was found to be 1.06, 1.09, and 1.08 for Newman, Wood46 and ATCC 55804 WTA analysis, respectively (Supplementary Table S1). Proton nuclear magnetic resonance spectra (Fig. 1A) were consistent with a 1,5-poly(ribitol phosphate) polymer in which the ribitol had been substituted by N-acetyl-D-glucosamine (GlcNAc). In accordance with previous reports^19,32^, the chemical shifts of the GlcNAc anomeric proton showed that WTAs of the Newman and Wood 46 strains were substituted at the O-4 position of ribitol with α-GlcNAc (5.07 ppm) and β-GlcNAc (4.75 ppm), respectively. As reported by Fattom *et al*.^18^, the proton NMR spectra of β-GlcNAc(1–4) WTA of the Wood 46 and ATCC 55804 strains were similar and showed a major difference in the chemical shifts of their respective anomeric proton (4.75 ppm for Wood 46 versus 4.66 ppm for ATCC55804; upfield shift of 0.09 ppm), indicating that the ATCC 55804 WTA (Ag336) was substituted with β-GlcNAc (4.66 ppm) at the O-3 position of the ribitol residue.

To further obtain quick and efficient structural information on WTAs of several strains, an HPAEC-PAD method was developed. The three purified WTAs were subjected to aqueous hydrogen fluoride (HF) treatment. This resulted in quantitative hydrolysis of phosphodiester bonds in the polysaccharides and the release of GlcNAc-Ribitol disaccharides, which could then be separated on a Carbopac MA1 column using HPAEC-PAD. Each disaccharide differing in GlcNAc glycosidic linkage eluted at a specific retention time: β-D-GlcNAc-(1→4)-ribitol at 12.1 min, β-D-GlcNAc-(1→3)-ribitol at 12.8 min and α-D-GlcNAc-(1→4)-ribitol at 13.5 min (Fig. 1B), along with some GlcNAc and ribitol residues due to acid-lability of the glycosidic bond in the GlcNAc-ribitol moiety in 48% HF^33^. The ribitol peak area was higher in the chromatograms of β-D-anomers of WTAs as β-D-anomers hydrolyzed more rapidly than α-D-anomers, as reported by Jennings and Lugowski^33^.

In addition, the HPAEC-PAD method was used to evaluate the proportion of α-GlcNAc(1–4) and β-GlcNAc(1–4) WTAs produced. The proportion was determined from the peak area of each structure relative to the sum of peak areas of all structures detected in the chromatogram (see formulas in Supplementary Methods). The consistency of the method was assessed from 5 independent experiments with purified WTAs of the Newman strain, which revealed to produce 98% and 2% of α-GlcNAc(1–4) and β-GlcNAc(1–4) WTAs, respectively. The Relative Standard Deviation was found at 0.5% for the α-GlcNAc(1–4) WTA (Supplementary Fig. S1). The accuracy of the method was further confirmed by proton NMR analyses of purified WTAs from 4 strains representative of the various proportions of WTAs found in our strain collection. The percentages of α-GlcNAc(1–4) and β-GlcNAc(1–4) WTAs were determined from the integration values of the anomeric protons and found to be similar to HPAEC-PAD results (Supplementary Figs S2 and S3). Therefore, although ribitol residue could be seen on chromatograms, the amount of released ribitol was negligible and had no impact on the calculation of α- and β-GlcNAc anomers.

The purified WTAs of the Newman, Wood 46 and ATCC55804 strains were further used as reference WTA samples for carbotyping of the other panel *S*. *aureus* strains.

### A majority of the *Staphylococcus aureu*s strains tested produces β-GlcNAc(1-4) WTA

**Figure 2:**
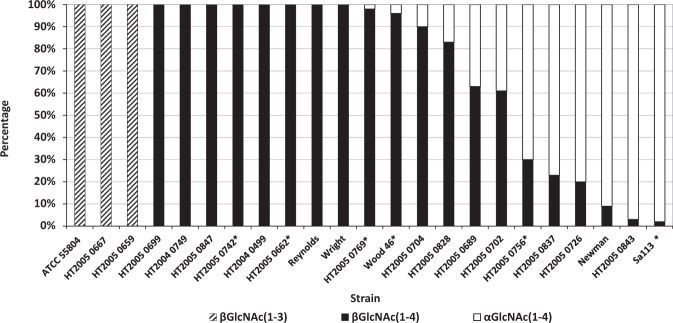
Distribution of WTA structures determined by HPAEC-PAD carbotyping of a panel of 24 *S*. *aureus* strains grown in TSB. The structures were determined directly from cell growth. The proportion of WTA structures in each strain, calculated as percentage, was determined from a single or two independent experiments for 18 and 6 strains, respectively, as indicated by an asterisk. For the 6 strains, the average percentage values are represented. The percentage was calculated from peak areas using the formulas described in the Supplementary Methods.

The structure of WTAs from the panel of 24 *S*. *aureus* strains was determined by HPAEC-PAD carbotyping using HF-treated bacteria grown on TSB and the proportion of α- and β-GlcNAc WTAs was calculated as described above and in Supplementary Methods. Two independent experiments were performed for 6 out of the 24 strains tested (Supplementary Fig. S4); for the remaining 18 strains only single experiments were performed. Structural variations were observed in WTAs but the majority of strains produced β-GlcNAc WTA (Fig. 2) on TSB. Across the 13 strains possessing only the *tarS* gene (Table 1), 10 strains exclusively substituted the hydroxyl at position 4 of the ribitol residue with β-GlcNAc while three strains substituted the hydroxyl at position 3 with β-GlcNAc. In the 11 other strains, which possessed both *tarS* and *tarM* genes, a mix of both structures was found in nine strains in various proportion, but with higher relative proportions of β-GlcNAc WTAs in four strains. Only two strains produced α-GlcNAc WTAs exclusively.

### α- and β-glycosylation of *S*. *aureus* WTA depends on growth media

**Figure 3:**
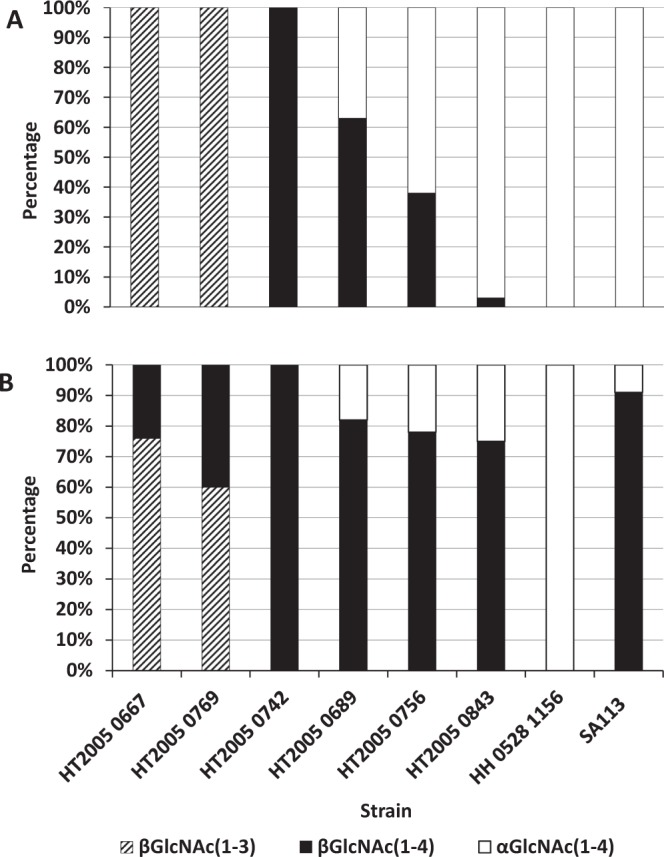
Comparison of WTA structure distribution of 8 *S*. *aureus* strains grown in (**A**) TSB and (**B**) SATA media. The structures were determined by HPAEC-PAD carbotyping directly from cell growth in the same HPAEC-PAD analysis. The proportion of WTA structures in each strain (calculated as percentage) was determined from single experiments and calculated from peak areas using the formulas described in the Supplementary Methods.

Eight strains representative of each *S*. *aureus* WTA glycosylation pattern and for the presence (five strains) or absence (three strains) of the *tarM* gene were selected and grown on high-NaCl-containing growth medium (SATA-2)^34^. The structure of WTAs was determined as previously described and compared to that of bacteria grown under normal conditions (TSB). Modification of the glycosylation pattern was observed for six strains (Fig. 3). Out of the five strains possessing both the *tarM* and *tarS* genes, four strains had up to 75–90% increased proportion of β-GlcNAc WTA. Interestingly, the HT2005 0667 and HT2005 0769 strains, which produced exclusively β-GlcNAc-glycosylated WTA at position 3 of the ribitol residue when grown in TSB, switched to a mix of WTAs glycosylated with β-GlcNAc glycosylation either at position 3 or 4 when grown on SATA-2.

### The Newman strain switches from α-GlcNAc WTA glycosylation *in vitro* to β-glycosylation *in vivo*

To assess whether structural changes in WTAs also occurred *in vivo*, two mouse infection models were carried out: the peritonitis model and the deep wound infection model. The Newman strain was used as a prototype strain, as the strain has been shown to produce almost exclusively α-GlcNAc WTA (>90%) under *in vitro* normal growth conditions despite the presence of both the *tarM* and *tarS* genes in the strain (Table 1; Fig. 2). In the peritonitis model, a group of five mice was infected by intraperitoneal route with a non-lethal dose of the Newman strain. Another group of five mice was infected with the HT2005 0742 strain as a control strain producing β-GlcNAc WTA, regardless the *in vitro* growth conditions (Fig. 3). In the deep wound infection model, one group of five mice was infected with the Newman strain.

Bacterial inocula were obtained from cultures grown on TSB. The structures were analyzed before infection and proven to be exclusively β-GlcNAc WTA in the HT2005 0742 strain, or predominantly α-GlcNAc WTA (>90%) in the Newman strain.

**Figure 4:**
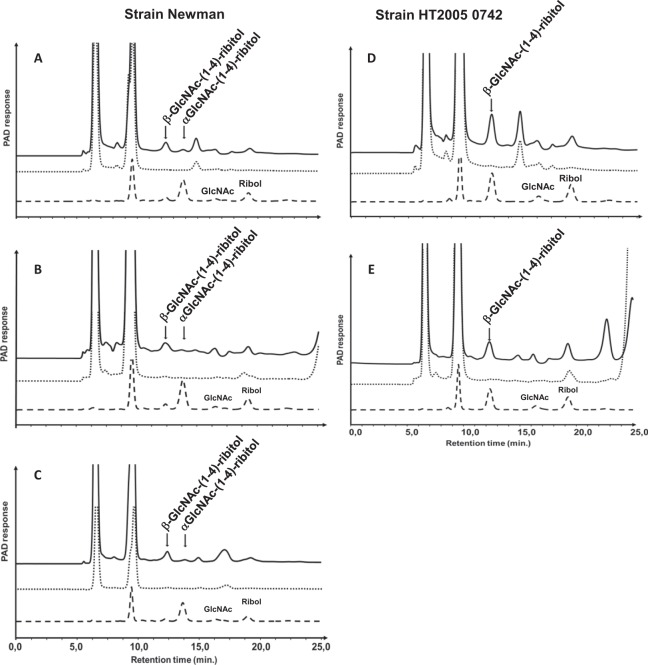
HPAEC-PAD analyses of WTA from infected mouse organs: kidneys (**A**,**D**), livers (**B**,**E**), and muscles (**C**). Chromatograms of GlcNAc-ribitol disaccharides obtained from mouse infected (solid line) with the *S*. *aureus* Newman (**A**–**C**) and HT2005 742 (**D**,**E**) strains compared to chromatograms of disaccharides obtained from the strains grown in TSB and used for mouse infection (dashed line). Samples from kidney of naïve mice were analyzed in parallel as negative reference (dotted line).

The structure of WTAs was determined by HPAEC-PAD directly on infected tissues without any WTA purification or bacteria culture step from infected organs. Figure 4 shows representative results obtained for each infected organ and each strain. In infected kidneys and livers from the peritonitis model, there was no change in the WTA structure of the HT2005 0742 strain, which retained the β-anomer, in agreement with the presence of the *tarS* gene only in this strain. In contrast, the WTA structure changed from the α- to the β-GlcNAc anomer in the Newman strain indicating that expression of the later was favored *in vivo* (Fig. 4). The expression of the β-GlcNAc anomer in the Newman strain was also demonstrated in infected muscles from the deep wound model.

## Discussion

The WTAs of most *S*. *aureus* strains are polysaccharides containing 11–40 ribitol phosphate repeat units with the ribitol residue substituted with D-alanine and GlcNAc at the O-2 and O-4 position, respectively. Earlier studies had revealed strain-specific pattern of α- and β-GlcNAc substitution at position O-4 of the ribitol residue^13–15^. Interestingly, WTAs isolated from the ATCC 55804 and N315 strains were found to display β-GlcNAc glycosylation at the O-3 position of the ribitol residue^18,35^ (the present study). An alternative glycosyltransferase, TarP, was recently identified to be responsible for this β-GlcNAc glycosylation^35^.

The N-acetylglucosaminyl-ribitol linkage of WTA is an immunological determinant in host immune responses; the specificity of elicited antibodies is dependent on the α- or β-GlcNAc anomeric form^36^ and possibly on the position in the ribitol residue. To design an efficient WTA vaccine antigen, it is crucial to determine the exact WTA glycosylation pattern of a diverse range of staphylococcal strains responsible for human infections, in order to identify the most representative structure so as to ensure the broadest coverage among invasive strains. To date, the distribution of the various WTA structures from a diverse panel of invasive *S*. *aureus* strains has never been reported.

In the present study, we selected 24 *S*. *aureus* strains representative of clinical isolates. The strains were characterized for the presence of genes encoding the two TarM and TarS glycosyltansferases responsible for modifying WTA with α-GlcNAc and β-GlcNAc respectively^19,20^. The *tarS* gene was found in almost all strains, while *tarM* was found in less than 50% of the strains but always in association with *tarS*. This is in agreement with previously reported distribution of *tarS* and *tarM* on two different panel of strains, where *tarS* was absent on only one strain and *tarM* present on 36%^24^ or 55% of the strains studied^21^, respectively. In accordance with the presence of the *tarS* gene in all strains studied, the β-GlcNAc WTA anomeric form was predominant in *S*. *aureus* clinical isolates. Brown *et al*.^20^ reported that *tarS* but not *tarM* expression levels were strongly upregulated by oxacillin treatment, which suggest a role for TarS in β-lactam resistance and highlights the importance of growth environment on gene expression. Our results provide further evidence of environmental influence on α- and β-glycosylation. We have demonstrated on eight strains that *in vitro* stress-inducing growth conditions (SATA-2 medium containing high NaCl concentration) favor the production of the β-GlcNAc anomer at the expense of the α anomer. Out of the 5 strains displaying both *tarM* and *tarS* genes, only one strain (HH0528 1156 strain) did not modify its glycosylation pattern and  kept producing exclusively α-GlcNAc WTA. Full genome sequencing was performed on this strain and indicated that both *tarS* and *tarM* gene were not mutated and had the potential to express functional enzymes. Our data thus indicate that genetic characterization alone may not accurately reflect the phenotype of WTAs and underline the importance of determining the actual glycotype.

The β-glycosylation of WTAs has been reported at either the O-3 position or the O-4 position 4 of ribitol^4,35^. We identified three strains (12.5% of the strain tested) that glycosylated exclusively WTA at position 3 when grown in a complex, commercial medium (TSB). Two of these strains (HT2005 0667 and HT2005 0769) were also grown on SATA-2 and interestingly, while the *tarP* gene has been found dominant over *tarS*^35^, their glycosylation pattern was modified to β-GlcNAc glycosylation at both position 3 and 4. Our study demonstrates that *S*. *aureus* can modulate the relative amounts of α- and β-glycosylation depending on environmental conditions, as well as the position of β-GlcNAc on ribitol. However, the regulation system that controls the full WTA glycoprofile remains to be determined. By *in silico* genome scanning, *tarM* was identified as part of the two-component GraRS regulon and to be positively regulated by this system *in vivo*^37^. Although GraRS, was shown to sense and confer resistance to selected cationic antimicrobial peptides^38^, it was also suggested that this system may respond to other signals like oxidative stress^37^. While the structure of TarS and TarM have been fully elucidated^39,40^, their gene regulation remains to our knowledge elusive. Of note, it has been reported that *tarS* but not *tarM* expression was strongly upregulated by oxacillin (β-lactam) treatment^20^.

Recent studies have emphasized the biological significance of the β-GlcNAc WTA anomeric configuration over the α-GlcNAc, and have provided indirect indications of the *in vivo* preferential selection of the β-form^22,41,42^. However, none of these studies directly assessed the structure and the diversity of the WTAs expressed. Human sera contain high levels of antibodies directed against *S*. *aureus* WTA^22,42–44^. Anti-β-GlcNAc WTA-IgG level is higher than that of anti-α-GlcNAc WTA-IgG in pooled human IgG fractions and in intact sera from healthy adults and infants^22^ suggesting that the β-GlcNAc anomer is an immunodominant antigen in staphylococcal infections. More specifically, β-GlcNAc WTA residues are required for induction of anti-WTA IgG-mediated C3 deposition and opsonophagocytosis^22,41^. Thus, Kurokowa *et al*.^23^ hypothesized that β-GlcNAc WTA might be more antigenic than α-GlcNAc WTA or that the β form may be more stable than the α form *in vivo*.

In the present study, we report for the first time the characterization of WTA directly on infected mouse tissues without any purification or bacteria culture step. Similar to what is observed in humans, *S*. *aureus* can induce a diverse spectrum of diseases in mice^45^ and therefore, two mouse infection models were used. Our study provides direct evidence that *in vivo* environmental conditions lead preferentially to β-glycosylation of the ribitol residue as stress-inducing culture conditions (high NaCl concentration) does *in vitro*. The upregulation of β-GlcNAc anomer in the Newman strain was observed in the peritonitis (livers and kidneys) and skin-wound infection (quadriceps muscles) models. While this strain displayed both functional TarS and TarM, only β-glycosylated WTAs were recovered from mice infected organs. Those results are consistent with reports showing that the level of anti-β-GlcNAc WTA antibodies is higher than that of anti-α-GlcNAc WTA antibodies in human sera^22^.

Dorling *et al*.^46^ has proposed that WTA itself would be involved in the evasion of immune recognition, while D-alanylation of WTA would be involved in mediating infection persistence. D-alanylation of TA is decreased when *S*. *aureus* are grown in medium containing high NaCl concentration due to the transcriptional repression of the *dltABC* operon^47^. Therefore, the decrease of D-alanylation may lead to a decrease of *S*. *aureus* resistance to cationic antimicrobial peptides. In that context, *S*. *aureus* may upregulates WTA β-glycosylation to overcome the decrease of infection persistence. Indeed, β-glycosylation of WTA is critical for the resistance of *S*. *aureus* MRSA strains to β-lactam^20^ and WTA linkage to peptidoglycan (PG) contributes to the resistance of *S*. *aureus* to lysozyme^48^. These observations support the hypothesis that β-glycosylated WTA could sterically hindered PG, preventing its enzymatic hydrolysis and release of its fragments. Furthermore, heterogeneous Vancomycin-Intermediate *S*. *aureus* (hVISA) and MRSA strains are more resistant than Methicilline Sensitive *S*. *aureus* (MSSA) strains to opsonophagocytosis and killing by phagocytes in the presence of low concentrations of serum^49^. Although a role of capsular polysaccharide that has been previously shown to prevent nonspecific killing of *S*. *aureus* cannot be excluded^50^, a molecular epidemiology study conducted from 91 *S*. *aureus* isolates from 2004 to 2005 showed that the majority of MRSA isolates, including the most prevalent Community Acquired-MRSA clone, USA300, were unencapsulated^51^. We suggest that resistance to opsonophagocytosis in MRSA strains may be primarily related to WTA β-glycosylation. In addition, Gautan *et al*.^52^ recently reported that WTAs serve as a barrier against opsonin recruitment to the cell wall and contribute to repulsion of peptidoglycan-targeted antibodies. Thus, we propose that expression of WTA β-glycosylation is one of the immune evasion strategies of *S*. *aureus* to resist to immune host defense.

In conclusion, the present study provides significant insight into the structural glycosylation diversity of WTAs among *S*. *aureus* strains, and demonstrates environmental influence in α- and β-glycosylation of WTAs both *in vitro* and *in vivo*. These findings, taken together with previous reports^20,22,49,52^ suggest that *S*. *aureus* with β-GlcNAc WTA may provide a competitive advantage during infection and support β-GlcNAc WTA as an appropriate target for vaccine-based immunotherapy/prophylaxis against invasive *S*. *aureus* infections.

## Materials and Methods

### Bacterial strains, growth conditions and genetic characterization

Overall 24 *S*. *aureus* strains were included in the study (Table 1). Most of them (18 strains) were 2005 clinical isolates (HT) from the French National reference Center for Staphylococci (Lyon, France) and were kindly provided by Prof Jérome Etienne (Hôpital Edouard Herriot, Lyon, France) along with their *agr* allele, capsular and methicillin resistance genotypes characterization^34^. Seven prototype strains were also included from three different sources: American type Culture Collection (ATCC), Institut Pasteur collection (CIP) and NIAID repository. The presence or absence of *S*. *aureus* WTA glycosyltransferase genes *tarS* and *tarM* was verified by PCR using gene-specific primers as described previously^24^. The sequence of *tar*S and *tarM* genes were determined for strains HH0528 1156, HT2005 0756, HT2005 0843. DNA was extracted from colonies grown overnight on TSB agar (Difco BD, Pont de Claix, France) by using the GenElute™ Bacterial Genomic DNA Kit Protocol (NA2110-1KT; Sigma-Aldrich, St Quentin Fallavier, France) in presence of Lysostaphin (L7386; Sigma-Aldrich, St Quentin Fallavier, France). The quantity and quality of the genomic DNA were measured with the Nanodrop Spectrophotometer (Thermo Scientific, Waltham, MA), Qubit 3.0 Fluorometer (Life Technologies, Thermo Fischer Scientific) and Dropsense (Trinean, Gentbrugge, Belgium), and by gel electrophoresis. 1 ng of gDNA was used for preparing library with the Nextera XT DNA Library Preparation kit (FC-131-1024-Illumina) containing specific indexes for each library. Briefly, DNA was simultaneously fragmented and tagged with sequencing adapters, followed by an end repair, an A-tailing and the ligation of adaptors containing indexes. After a short amplification step, libraries were purified and then sequenced. The quantity and quality of the libraries were measured with the Qubit 3.0 Fluorometer (Life Technologies) and 2100 bioanalyzer System (Agilent, Santa Clara, CA). Sequencing was performed on MiSeq sequencer (Illumina, San Diego, CA) with the v2 Reagent Kit (MS-102-2002; Illumina) to generate 2 × 150 bp paired-end reads in a batch of 4 samples on the flow-cell. Raw data Quality Control was performed with FastQC (Babraham Bioinformatics, Cambridge, UK). The reads were trimmed and *de novo* assembled with CLCGenomics Workbench (v8.5; Qiagen, Redwood City CA). Blast was used to retrieve *tarS* gene sequence in the assembled contigs.

For teichoic acid analysis, bacterial strains were grown either in tryptic soy broth (TSB, Difco), or proprietary medium SATA-2 (93 g/L wheat peptone, 0.25 g/L D-glucose, 41 g/L NaCl and 15 g/L MgCl_2_) initially developed to increase capsular expression through high concentration of NaCl^34^.

### WTA purification and characterization

WTAs were extracted and purified from the Newman, Wood 46 and ATCC55804 strains grown in 500-mL culture of TSB for 24 h at 37 °C. The cultures were inactivated by treatment with phenol-ethanol (1:1, v/v) to a final concentration of 2%. The cells were collected by centrifugation at 5,000 × g for 1 hour at 4 °C and suspended in 0.05 M Tris, 2 mM MgSO_4_ pH 7.5 (0.5 g wet weight/mL). The cell suspensions were incubated with lysostaphin (100 µg/mL) at 37 °C for 3 hours with continuous stirring. MgCl_2_ and benzonase were subsequently added to a final concentration of 1 mM and 50 UI/mL, respectively, and incubated at 37 °C for 4 hours. The final concentration of Tris buffer was adjusted to 50 mM, and CaCl_2_ and pronase added to a final concentration of 1 mM and 0.5 mg/mL, respectively. Finally, the samples were incubated for 16 hours at 37 °C. The remaining insoluble cell debris were removed by centrifugation at 8,000 × g for 30 min. The supernatants were precipitated with 25% ethanol in presence of 10 mM CaCl_2_ and stirred for 16 hours at 4 °C. The precipitates were removed by centrifugation at 8,000 × g for 30 min and the supernatants containing WTAs were precipitated with 75% ethanol in presence of 10 mM CaCl_2_ and stirred for 4 hours at 4 °C, collected by centrifugation at 8,000 × g for 30 minutes and dissolved in water. The samples were dialyzed extensively against water at room temperature. 1 M Tris buffer pH 7.0 was added to a final concentration of 50 mM and loaded onto a Q Sepharose column (GE Healthcare, Uppsala Sweden). WTAs were separated from residuals using a linear gradient 0–0.5 M NaCl in 50 mM Tris buffer pH 7.0. Fractions containing WTA as detected by the modified Elson-Morgan hexosamine assay^53^ were pooled, extensively dialyzed against water at room temperature and freeze-dried.

Monosaccharide composition of WTAs was determined using High-Performance Anion-Exchange Chromatography with Pulsed Amperometry Detection (HPAEC-PAD) (Thermo Fischer Scientific, Dionex, Sunnyval, CA) as previously described^54^ and detailed in Supplementary Methods. Proton nuclear magnetic resonance (^1^H NMR) spectra of WTAs were recorded using a 500-MHz Bruker Avance DRX spectrometer (Bruker Biospin, Wissembourg, France) at 293 K in D_2_O.

### Mouse infection models with *S*. *aureus*

Female outbred OF1 mice were obtained from Charles River Laboratories (Saint-Germain-sur-l’Arbresle, France). All mouse procedures were performed under general anesthesia. Animals were housed and handled according to European regulations. The procedures were reviewed and approved by the Sanofi Pasteur animal care committee. Bacterial suspensions were obtained from 50-mL cultures of the Newman or HT2005 0742 strains grown for 20 hours in TSB at 37 °C.

### Peritonitis model

Mice were infected by the intraperitoneal route with 1.7 × 10^6^ CFU/500 µL of the Newman strain or 7.0 × 10^6^ CFU/500 µL of the HT2005 0742 strain. Bacterial inocula were prepared extemporaneously by mixing 1:1 sterile 20% hog mucin and 2x concentrated adjusted bacterial suspensions. The mice were euthanized 15 days post-infection. Livers and kidneys were removed.

### Deep wound model

The hair from the left thighs was shaved and the area disinfected. An incision measuring 1 cm in length was carried through the skin. The incision was then continued to a depth of 0.5 cm and 0.4 cm in depth into the underlying quadriceps muscles. The muscle incisions were closed with one silk suture and the wounds were inoculated under the suture with 2.5 µL of a Newman *S*. *aureus* suspension containing 10^3^ CFU. Finally, the skin incisions were closed with two separated prolene sutures. The mice were treated with 70 µL/20 g Buprecare (Alcyon, Paris, France) injected by intraperitoneal route on a regular basis. Clinical evidence of wound infection, defined as the presence of an abscess and purulent infection within the wound, was observed in all animals two days after the infection. The mice were euthanized three days post-infection. Quadricep muscles were removed.

Infected organs were obtained 15 days and 3 days post-infection in the peritonitis and deep wound models respectively then dissociated and homogenized in sterile phosphate buffered saline (PBS) under aseptic conditions for direct analysis of WTA structure.

### WTA carbotyping by HPAEC-PAD from cell growth and infected organs

10^9^ CFUs of *S*. *aureus* grown either in TSB or SATA-2 medium until stationary phase were collected by centrifugation at 5,000 × g at 4 °C for 20 min and washed with 0.5 mL of 0.15 M NaCl. Preliminary experiments have showed that the minimal amount of CFU required for the direct detection of WTA in infected organs by HPAEC-PAD is 7 log_10_ total CFU. Therefore, mouse kidneys, livers and muscles containing more than 7 log_10_ total CFU were ground and washed twice with 5 mL, 1 mL and 0.5 mL of 0.15 M NaCl, respectively.

The samples containing either the bacterial cells or the ground organs were suspended in 400 µL of aqueous hydrofluoric acid (HF) (48% by mass) and incubated at room temperature overnight. Cell debris were removed by centrifugation and acid removed under a stream of nitrogen at 40 °C. The samples were dissolved in 400 µL of water and passed through a centrifugal filter unit (10 kDa MW cut-off, Ultracel-10, Millipore) to remove proteins and other macromolecules. The disaccharides generated by HF hydrolysis were separated on a Dionex system using a CarboPac MA1 (4 mm × 250 mm) analytical column with a guard column (4 mm × 50 mm) previously equilibrated in 480 mM NaOH at a flow rate of 0.4 ml/min. The disaccharides were separated isocratically using 480 mM NaOH for 40 min. Purified and characterized WTAs from the Newman, Wood46 and ATCC55804 strains were hydrolyzed in the same way and used as references for peak assignement. The proportion of each WTA structure in purified WTAs or strains were calculated as described in Supplementary Methods.

## Supplementary information

Glycosylation of Staphylococcus aureus cell wall teichoic acid is influenced by environmental conditions
