# Supplementary material for: Glycosylation of Staphylococcus aureus cell wall teichoic acid is influenced by environmental conditions
Source: Sci Rep. 2019 Mar 1;9:3212. doi: 10.1038/s41598-019-39929-1 (PMC6397182; doi:10.1038/s41598-019-39929-1)
Supplement: Supplementary file 1 — Glycosylation of Staphylococcus aureus cell wall teichoic acid is influenced by environmental conditions [file 41598_2019_39929_MOESM1_ESM.docx]

**Supplementary Information**

**Glycosylation of *Staphylococcus aureus* cell wall teichoic acid is influenced by environmental conditions**

Noëlle Mistretta*, Marina Brossaud, Fabienne Telles, Violette Sanchez, Philippe Talaga and Bachra Rokbi

*Research and Development, Sanofi Pasteur, Marcy l’Etoile, France*

*** Corresponding author**

Noëlle Mistretta, Research and Development, Sanofi Pasteur, 1541 Avenue Marcel Mérieux, 69280 Marcy l’Etoile, France

Tel: +33 4 37 37 38 68; E-mail: noelle.mistretta@sanofi.com

**Supplementary Methods**

**Compositional Analysis by High-Performance Anion-Exchange Chromatography with Pulsed Amperometric Detection (HPAEC-PAD).** Samples (triplicates from 1 to 4 µg freeze-dried WTAs in 400 µL water) were transferred to a polypropylene microfuge tube, dried under a stream of nitrogen, and 200 μL of 48% (v/v) HF was added. After incubation at 4°C for 24 h, the acid was removed under a stream of nitrogen at ca. 40°C, redissolved in 400 µL of water, and transferred to a glass screw-cap. The 400-μL sample was mixed with 100 μL of a 10N TFA (final concentration 2N) in a glass tube sealed with a screw-cap. After 2 h at 121°C, the acid was removed under a stream of nitrogen and the residues were then redissolved in 400 μL of water and transferred to autosampler vials. Chromatography of the samples was performed using a Dionex ICS3000 ion chromatography system (Dionex, Sunnyvale, CA) coupled to an AS50 autosampler. Separations were performed on a CarboPac MA1 ( 4x250 mm) analytical column (Dionex) with a guard column (4x50 mm) using a 480 mM sodium hydroxide solution at a flow rate of 0.4 mL/min. Pulsed amperometric detection was used incorporating a quadruple-potential waveform. Data were collected and analyzed on computers equipped with Dionex Chromeleon Software (Dionex). Monosaccharide standards used were Ribitol (SIGMA ref A-5502 ) and Glucosamine (GlcN) (USP ref 1294207) as a mix. Linear calibration curves were obtained and used for quantification. The linear range extended from 0.6-10 nmol/mL for ribitol and from 0.5-20 nmol/mL for GlcN.

**Table S1**: **Molar composition of purified WTAs from the Newman, Wood 46 and ATCC 55804 strains**. Samples S1, S2 and S3 are triplicates realized in the same experiments.

| **Strain** | **Sample#** | **GlcN:Ribitol molar ratio** | **Mean** | **Standard deviation** | **RSD** |
| --- | --- | --- | --- | --- | --- |
| Newman | S1 | 1.06 | 1.06 | 0.01 | 1% |
|  | S2 | 1.08 |  |  |  |
|  | S3 | 1.05 |  |  |  |
| Wood 46 | S1 | 0.99 | 1.09 | 0.07 | 7% |
|  | S2 | 1.14 |  |  |  |
|  | S3 | 1.14 |  |  |  |
| ATCC55804 | S1 | 1.04 | 1.08 | 0.05 | 5% |
|  | S2 | 1.04 |  |  |  |
|  | S3 | 1.15 |  |  |  |

**Carbotyping of purified WTAs by HPAEC-PAD.** Sample containing from 10-20 µg freeze-dried WTAs in 400 µL water were transferred to a polypropylene microfuge tube, dried under a stream of nitrogen, and 200 μL of 48% (v/v) HF was added. After incubation at room temperature overnight, the acid was removed under a stream of nitrogen at ca. 40°C, redissolved in 400 µL of water and transferred to a glass screw-cap.The disaccharides generated by HF hydrolysis were separated on a Dionex system using a CarboPac MA1 (4x250 mm) analytical column with a guard column (4x50 mm) previously equilibrated in 480 mM NaOH at a flow rate of 0.4 mL/min. The disaccharides were separated isocratically using 480 mM NaOH for 40 min. The proportion of each WTA structure in purified WTAs or strains was calculated from the disaccharide peak areas using the following formulas:

$$\% \mathrm{GlcNAc}\left( 1,3 \right)= \frac{GlcNAc(1,3) peak area}{\mathrm{GlcNAc}\left( 1,3 \right)peak area+ GlcNAc(1,4) peak area +GlcNAc(1,4) peak area} x 100\text{ (1)}$$

$$\% \mathrm{GlcNAc}\left( 1,4 \right)= \frac{GlcNAc(1,4) peak area}{\mathrm{GlcNAc}\left( 1,3 \right)peak area+ GlcNAc(1,4) peak area +GlcNAc(1,4) peak area} x 100\text{ (2)}$$

$$\% \mathrm{GlcNAc}\left( 1,4 \right)= \frac{GlcNAc(1,4) peak area}{\mathrm{GlcNAc}\left( 1,3 \right)peak area+ GlcNAc(1,4) peak area +GlcNAc(1,4) peak area} x 100 \text{(3)}$$

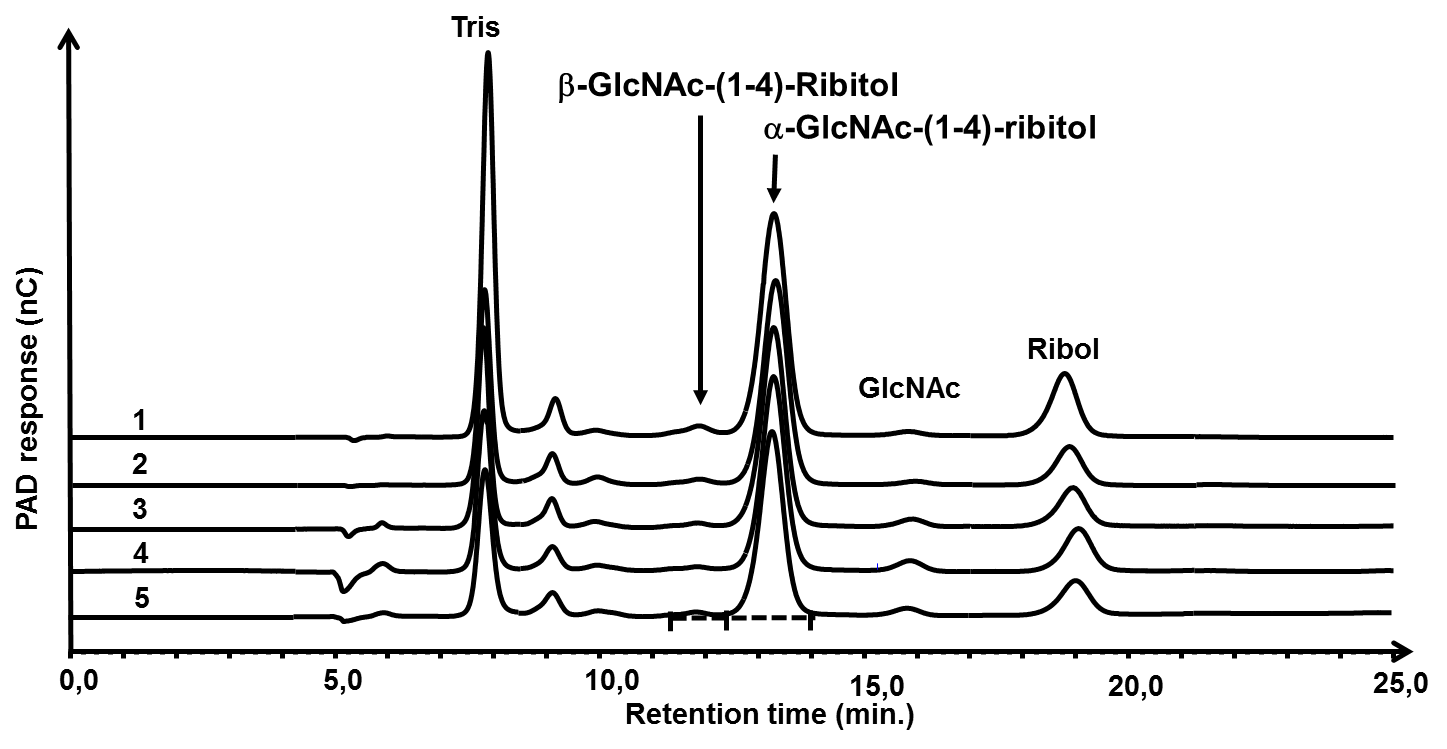


**B**

**A**

| **Experiment #** | **α-GlcNAc** | | **β-GlcNAc** | |
| --- | --- | --- | --- | --- |
|  | **Peak Area**  **nC*min** | **Proportion %** | **Peak Area**  **nC*min** | **Proportion %** |
| 1 | 124.39 | 96.9 | 3.98 | 3.1 |
| 2 | 78.42 | 97.8 | 1.80 | 2.2 |
| 3 | 61.49 | 98.1 | 1.19 | 1.9 |
| 4 | 24.05 | 98.2 | 0.44 | 1.8 |
| 5 | 160.47 | 97.5 | 4.09 | 2.5 |
| Average |  | 97.7 |  | 2.3 |
| RSD |  | 0.5% |  | 20.3% |

**Figure S1: HPAEC-PAD analyses of purified *S. aureus* Newman WTAs**. A) HPAEC-PAD chromatograms of HF-hydrolyzed WTAs obtained from 5 independent experiments. The chromatograms have been normalized on α-GlcNAc (1-4)- Ribitol disaccharide peak. B) Peak area and calculated proportion of α-GlcNAc and β-GlcNAc populations. RSD, relative standard deviation.


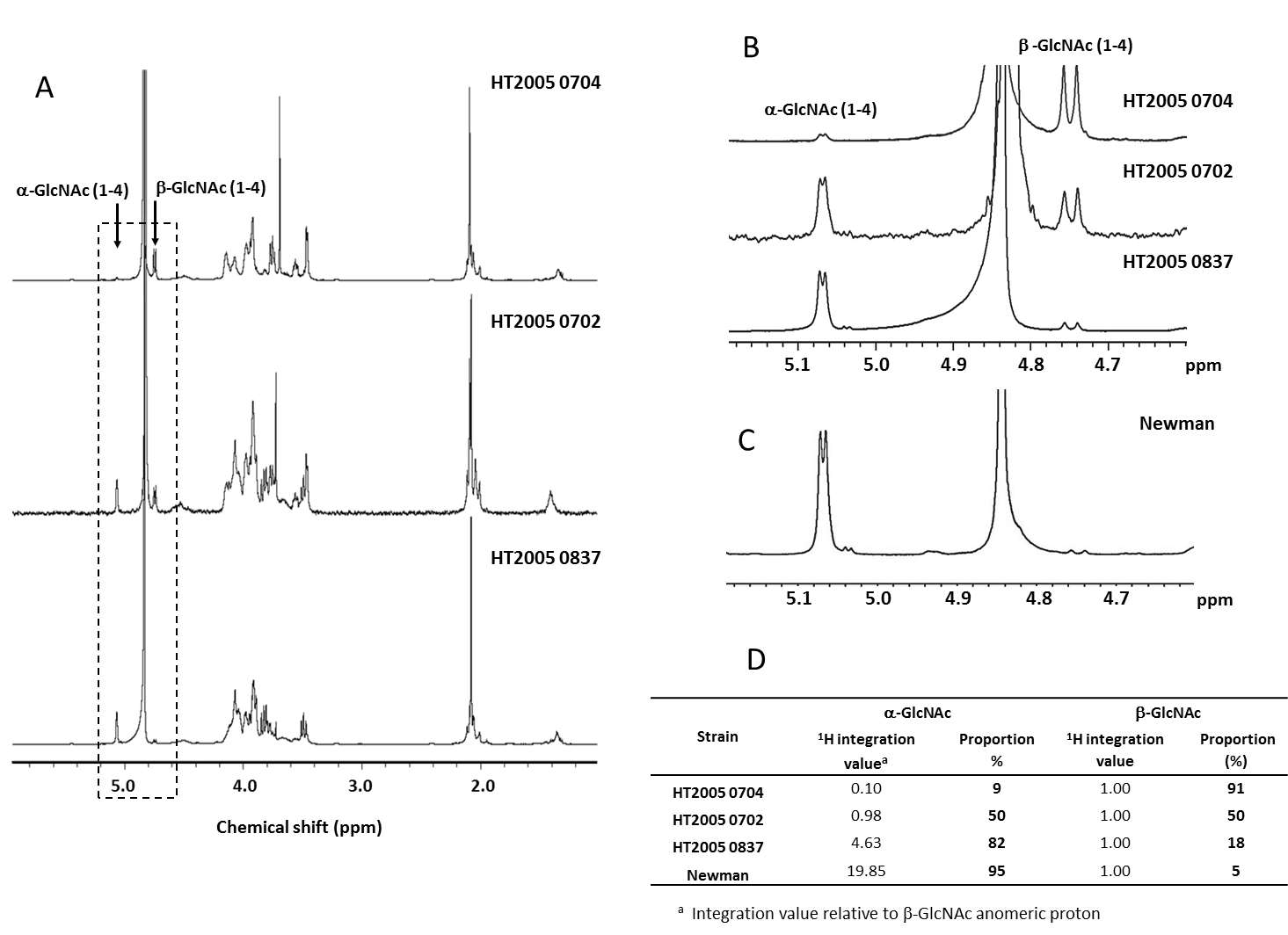


**Figure S2**: **500 Mz ^1^H NMR analyses of purified *S. aureus* WTAs**. A) ^1^H NMR spectra of WTAs from strains HT2005 0704, HT2005 0702 and HT2005 0837. Expansion of the anomeric region of B) HT2005 0704 and HT2005 0702 WTA and C) Newman WTA. D) Integration values of anomeric protons and calculated proportion of β-GlcNAc and α-GlcNAc populations in each WTA.

**
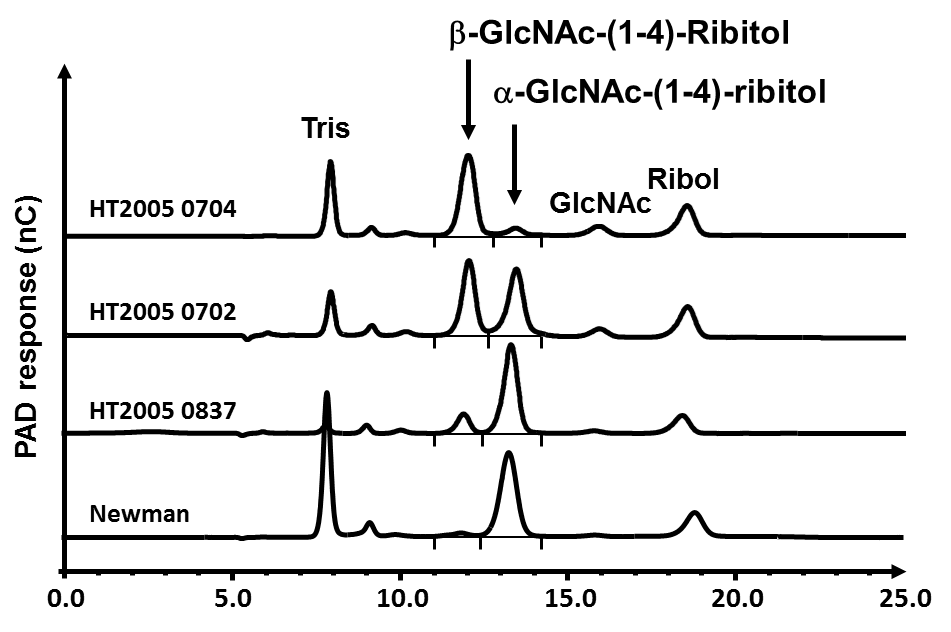
**

A

| **Strain** | **α-GlcNAc** | **β-GlcNAc** |
| --- | --- | --- |
|  | **Proportion %** | **Proportion %** |
| HT2005 0704 | 10 | 90 |
| HT2005 0702 | 52 | 48 |
| HT2005 0837 | 83 | 17 |
| Newman | 97 | 3 |

B

**Figure S3**: **HPAEC-PAD analyses of purified *S. aureus* WTAs.** A) HPAEC-PAD chromatogram of HF‑hydrolyzed purified WTAs from strains HT2005 0704, HT2005 0702, HT2005 0837 and Newman.
B) Proportion of α-GlcNAc and β-GlcNAc anomers in each WTA determined from peak areas as described in Supplementary Methods.

**
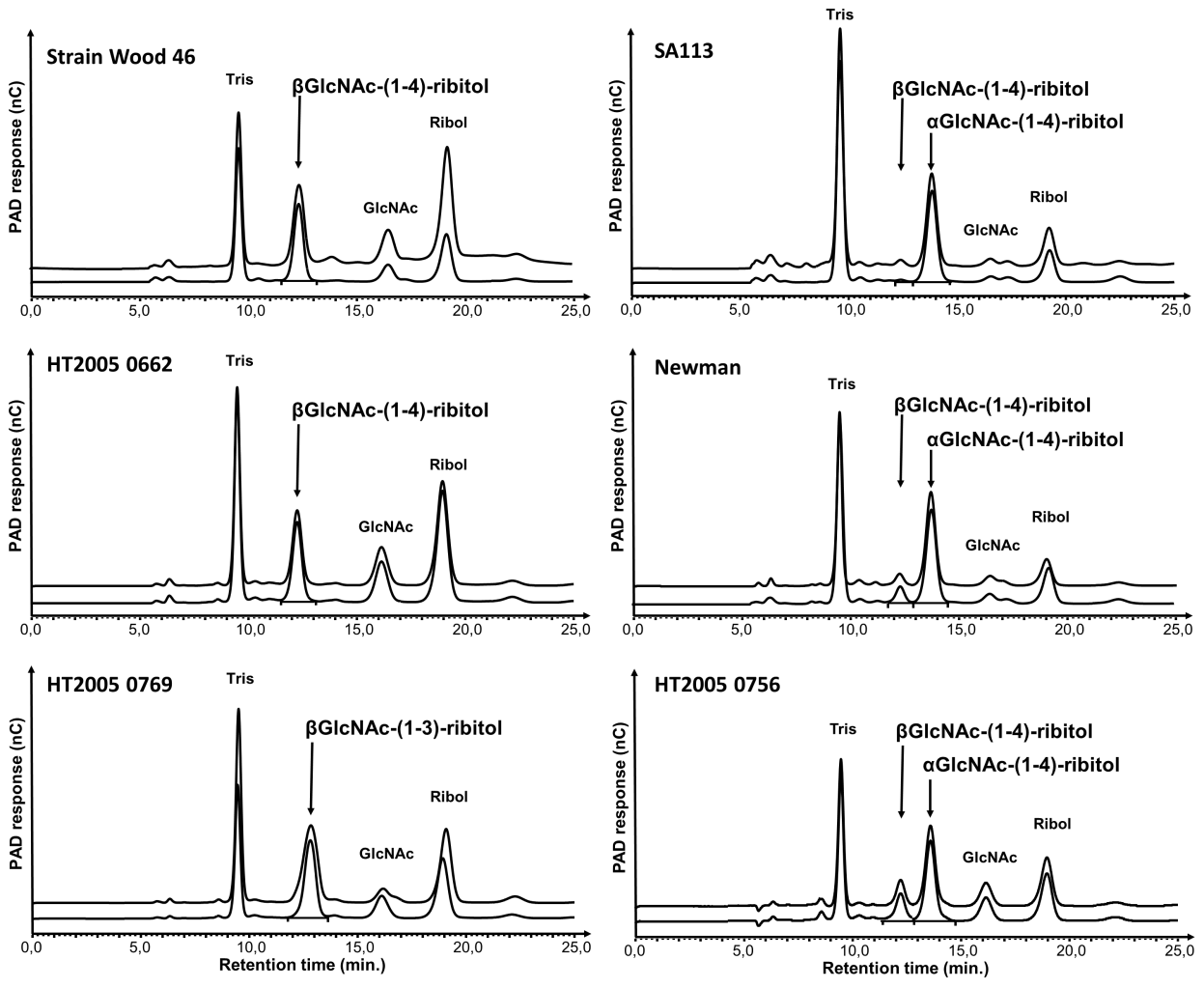
**

B

A

| **Strain** |  | **α-GlcNAc (1-4)** | | **β-GlcNAc-(1-4)** | | **β-GlcNAc-(1-3)** |  |
| --- | --- | --- | --- | --- | --- | --- | --- |
|  | **Experiment** | **Peak Area**  **nC*min** | **Proportion %** | **Peak Area**  **nC*min** | **Proportion %** | **Peak Area**  **nC*min** | **Proportion %** |
| **Wood46** | 1 | nd | - | 318.31 | 100 | nd | - |
|  | 2 | nd | - | 359.09 | 100 | nd | - |
|  | **Average** |  | **-** | **-** | **100** |  |  |
| **HT2005 0662** | 1 | nd | - | 101.24 | 100 | nd |  |
|  | 2 | nd | - | 150.92 | 100 | nd | - |
|  | **Average** |  | **-** | **-** | **100** |  | - |
| **HT2005 0769** | 1 | nd | - | nd | - | 198.28 | 100 |
|  | 2 | nd | - | nd | - | 169.42 | 100 |
|  | **Average** |  |  |  |  |  | **100** |
| **SA113** | 1 | 500.09 | 98 | 8.15 | 2 | nd | - |
|  | 2 | 242.98 | 94 | 15.3 | 6 | nd | - |
|  | **Average** |  | **96** |  | 4 |  |  |
| **Newman** | 1 | 484.42 | 88 | 67.32 | 12 | nd | - |
|  | 2 | 153.01 | 91 | 15.07 | 9 | nd | - |
|  | **Average** |  | **90** |  | **10** |  |  |
| **HT2005 0756** | 1 | 145.88 | 62 | 88.42 | 38 | nd | - |
|  | 2 | 33.01 | 77 | 10.05 | 23 | nd | - |
|  | **Average** |  | **70** |  | **30** |  |  |

nd: not detected

**Figure S4: HPAEC-PAD analyses of *S. aureus* WTAs from cell growth performed in two independent experiments.** A) HPAEC-PAD chromatogram of HF‑hydrolyzed cells; B) Proportion of α-GlcNAc and β-GlcNAc anomers in each WTA determined from peak areas as described in Supplementary Methods.
